# IoT Solutions and AI-Based Frameworks for Masked-Face and Face Recognition to Fight the COVID-19 Pandemic

**DOI:** 10.3390/s23167193

**Published:** 2023-08-15

**Authors:** Jamal Al-Nabulsi, Nidal Turab, Hamza Abu Owida, Bassam Al-Naami, Roberto De Fazio, Paolo Visconti

**Affiliations:** 1 Medical Engineering Department, Faculty of Engineering, Al-Ahliyya Amman University, Amman 19328, Jordan; j.nabulsi@ammanu.edu.jo (J.A.-N.); h.abuowida@ammanu.edu.jo (H.A.O.); 2 Department of Networks and Cyber Security, Faculty of Information Technology, Al-Ahliyya Amman University, Amman 19328, Jordan; n.turab@ammanu.edu.jo; 3 Department of Biomedical Engineering, Faculty of Engineering, The Hashemite University, Zarqa 13133, Jordan; b.naami@hu.edu.jo; 4 Department of Innovation Engineering, University of Salento, 73100 Lecce, Italy; roberto.defazio@unisalento.it

**Keywords:** face recognition, machine learning, deep learning, support vector machines, convolutional neural networks (CNN)

## Abstract

A global health emergency resulted from the COVID-19 epidemic. Image recognition techniques are a useful tool for limiting the spread of the pandemic; indeed, the World Health Organization (WHO) recommends the use of face masks in public places as a form of protection against contagion. Hence, innovative systems and algorithms were deployed to rapidly screen a large number of people with faces covered by masks. In this article, we analyze the current state of research and future directions in algorithms and systems for masked-face recognition. First, the paper discusses the importance and applications of facial and face mask recognition, introducing the main approaches. Afterward, we review the recent facial recognition frameworks and systems based on Convolution Neural Networks, deep learning, machine learning, and MobilNet techniques. In detail, we analyze and critically discuss recent scientific works and systems which employ machine learning (ML) and deep learning tools for promptly recognizing masked faces. Also, Internet of Things (IoT)-based sensors, implementing ML and DL algorithms, were described to keep track of the number of persons donning face masks and notify the proper authorities. Afterward, the main challenges and open issues that should be solved in future studies and systems are discussed. Finally, comparative analysis and discussion are reported, providing useful insights for outlining the next generation of face recognition systems.

## 1. Introduction

Since the 1990s, image recognition has become a prominent topic, exploiting artificial intelligence (AI) and technological advancements. Face and object recognition is a common technique in computer vision and arguably its most fundamental aspect [1,2].

Most facial and object detection systems rely on traditional machine learning (ML) techniques; a balance learning framework was proposed in [3] to enhance the training of the networks, resulting in improved performance than previous ones.

Public and private organizations utilize facial recognition technology to identify and regulate admission to airports, schools, and offices. Because of its apparent accuracy in identifying people, face recognition systems have been used in healthcare to improve patient condition information security, sanitation with contactless applications, staff access point security, and patient and worker data collection [4].

In the last year, masked-face recognition has expanded applicability thanks to Internet of Things (IoT) devices to recognize and identify individuals wearing masks. With the widespread adoption of face masks in response to the COVID-19 pandemic, traditional facial recognition systems that rely on full-face visibility have faced challenges in accurately identifying individuals [5]. Masked-face recognition in IoT involves combining IoT devices, such as surveillance cameras or smart doorbells, with specialized algorithms and technologies to recognize and verify individuals even when wearing masks [6]. Furthermore, different face mask recognition systems were deployed during the COVID-19 health emergency to verify whether individuals correctly wore masks in public places.

Challenges in masked-face recognition include dealing with variations in mask types, lighting conditions, and occlusions caused by masks. Technological advancements, such as improved algorithms and hardware, can help overcome these challenges and enhance the accuracy of masked-face recognition in IoT applications. However, it is important to consider privacy and ethical concerns associated with facial recognition technology and ensure proper safeguards are in place to protect individuals’ rights and data.

The development of numerous digital technologies over the past decade has made it possible to employ them to fight the COVID-19 pandemic [7]. Artificial intelligence (AI) has recently been used to improve the identification of infection levels, as well as to locate and diagnose illnesses quickly.

In the late 1990s, the Bochum system, which used a Gabor filter to store face data and generated a grid of the face structure to link the characteristics, supplanted purely feature-based systems for facial recognition [8]. In the mid-1990s, Elastic Bunch Graph Matching was developed using skin segmentation to recover a face from a photograph [9]. Elastic Bunch Graph Matching was created in the mid-1990s to recover a face from an image using skin segmentation [9]. The algorithm was stable enough to identify individuals from less-than-ideal face views. It can also see through hurdles to identification, such as mustaches, beards, new hairstyles, spectacles, and even sunglasses [9].

Real-time face identification from video became possible with the release of the Viola-Jones object detection framework for faces in 2001 [10]. Paul Viola and Michael Jones presented AdaBoost, the first real-time frontal-view face detector, by merging their face detection algorithm with the Haar-like feature approach to object recognition in digital images [11]. In 2015, the Viola-Jones algorithm was implemented on mobile devices and embedded systems using small, low-power detectors. As a result, the Viola-Jones method has enabled new possibilities in user interfaces and teleconferencing, as well as expanded the practical use of facial recognition systems [12].

Ukraine is identifying dead Russian personnel using the Clearview AI facial recognition platform developed in the United States. Ukraine has identified the relatives of 582 slain Russian servicemen after conducting 8600 searches. The Ukrainian army’s information technology volunteer unit uses such software to inform the families of dead soldiers about Russian actions in Ukraine [13]. A new generation of AI software tools has been implemented in the surveillance cameras of the Paris Metro to ensure that passengers are wearing masks.

Deep learning and computer vision techniques for detecting COVID-19 face masks can help predict pandemic incidence based on anonymized and unidentifiable statistics data. The face mask recognition models provide the following advantages [14]:

- Reduce the propagation of the COVID-19 pandemic.
- Precise improvement of the detection performance.
- High processing speed and seamless integration with surveillance cameras.
- They can be applied in schools, universities, and other institutions that monitor attendance using facial recognition. Consequently, administrators can readily determine if students, employees, and other visitors are wearing masks.

Research papers [15,16] show significant advances in deep learning toward entity detection and recognition in numerous application domains have occurred over time. The detection of objects with deep learning and the early detection of a congestion control warning system on a bridge’s footsteps were extensively discussed.

Image reconstruction and facial recognition are now the subject of several investigations. However, the classification process based on machine learning (ML) and deep learning (DL) includes several steps that result in a final prediction to infer the input class based on extracted features [17].

Classification methods may be categorized into two types: supervised methods and unsupervised methods. ML learns to classify new inputs using supervised methods based on original labeled data, typically used to construct prediction models. According to [18], unsupervised ML uses unlabeled data to develop a model that predicts incoming inputs based on clustering algorithms.

**Figure 1:**
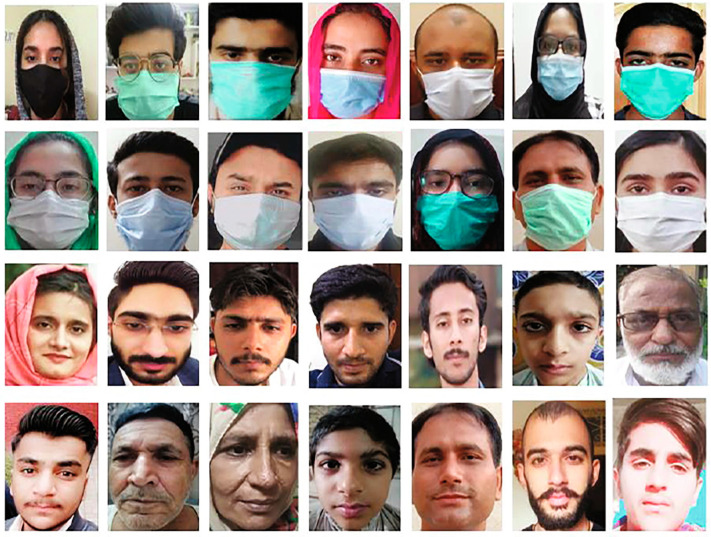
Sample images contained in the MDMFR dataset [19].

The DeepMasknet framework was proposed in [19]; it can be applied for face mask recognition and masked-face detection. Furthermore, as shown in Figure 1, the authors created a large and distinct integrated mask detection and masked facial recognition (MDMFR) dataset to evaluate the effectiveness of the proposed method.

The main contributions and novelties of the presented paper are as follows:

- A comprehensive overview of ML and DL algorithms and methods for face mask recognition and masked-face detection; in particular, operating modalities, advantages, and shortcomings of each method/algorithm are reported, along with application examples proposed in the scientific literature.
- A description of mobile networks for face mask detection and masked-face recognition applications developed during the COVID-19 pandemic to quickly detect whether people wear or not the mask, like Mobile Netv1 and MobileNetv2.
- A description of the main challenges and open issues for developing face mask detection systems, including precision, privacy, and improper use of private information.
- In-depth comparative analyzes of algorithms and models reported in the scientific literature to determine features and perspectives of innovative ML and DL tools for recognizing people wearing masks to fight future pandemics.

The remainder of the paper is organized as follows: in the following section, we discuss ML systems to recognize masked faces; then, Section 3 introduces DL methods for identifying masked faces, briefly discussing several scientific works and systems based on each algorithm and method. Furthermore, Section 4 presents examples of mobile networks developed for masked-face detection, like Mobile Netv1 and MobileNetv2. Moreover, IoT-based sensors for masked-face recognition were presented and developed during the COVID-19 pandemic. Section 6 reports challenges and open issues in developing systems and algorithms for masked-face recognition. Finally, Section 7 presents comparative analyses of systems and models reported in the scientific literature, comparing them from the performance perspective.

### Paper Selection and Bibliometric Indexes

**Figure 2:**
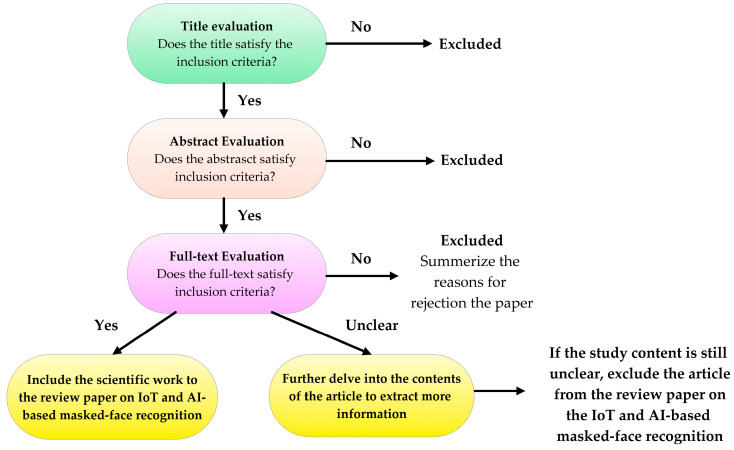
Workflow representing the inclusion/exclusion process for selecting the paper included in the presented review work.

An essential step in writing the presented review paper concerns the selection of discussed and analyzed papers according to set inclusion and exclusion rules. Detailed consideration of numerous characteristics of the studied documents, such as their applicability to the themes they address, their relevance, when they were published, and whether or not they overlapped with other chosen papers, was made while defining the latter. According to the procedure shown in Figure 2, the selection approach was carried out; a three-step analysis was completed, starting with the title, moving on to the abstract, and finishing with reading the whole text.

In this way, 67 scientific works were selected and included in the presented review paper, organized into 20 review papers, 42 articles, 4 book chapters, and 1 website.

**Figure 3:**
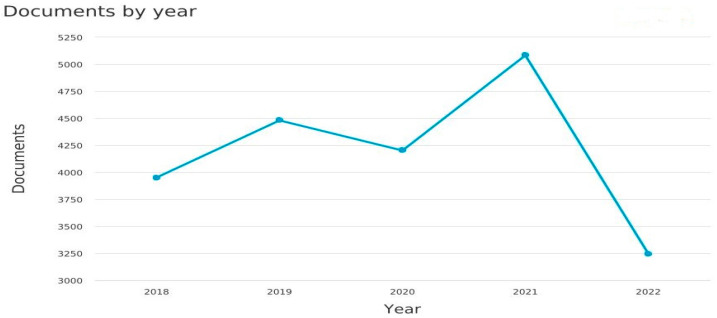
Trend of the face recognition-related documents by year.

**Figure 4:**
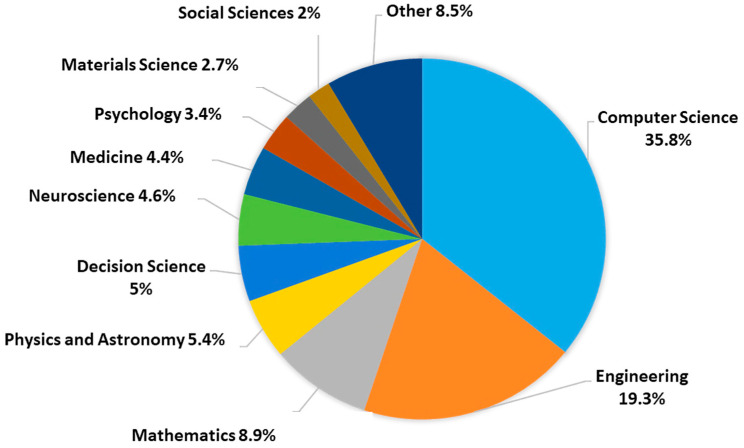
Face recognition-related documents classified by area.

Exploratory and bibliometric analysis was performed, using Scopus as a data source to investigate statistics related to scientific works on AI-based face recognition applications. Using the analysis tools provided by the Scopus platform, the obtained data were plotted in Figure 3 to illustrate that these topics have received more attention recently, particularly from 2018 to 2022. While Figure 4 explores publications that confronted computer science and engineering subject areas and other areas.

Referring to Figure 3, the highest number of documents was in 2021 (approximately 5084), beginning in 2018 with 3950 documents and terminating in 2022 with the lowest number (about 3247).

As shown in Figure 4, more than 35.8% of documents are in the computer sciences field, 19.35% are in the engineering field, and 44.9% are distributed across other disciplines.

## 2. Machine Learning Prototypes Used to Identify Face Mask

Machine learning is a branch of artificial intelligence (AI) and computer science that uses data and algorithms to mimic human learning processes and progressively increase accuracy. It can be used to identify relationships in a dataset through unsupervised, supervised, or hybrid learning. Supervised learning and unsupervised learning are the two basic approaches used in machine learning and artificial intelligence (AI). While supervised learning involves training a machine-learning model on labeled data, unsupervised learning involves training a machine-learning model on unlabeled data without predefined target labels or output values. Unsupervised learning aims to discover patterns, structures, or relationships within the data without explicit guidance. The two strategies differ in various ways, and there are some situations when one works better than the other. Using open-source and local data, ref. [20] summarizes the ML models and how they were trained.

Machine-learning algorithms can be used to identify face masks in various ways. Common approaches followed are [21,22]:

- Image classification: ML models can be trained to classify images based on whether a person is wearing a face mask or not. This approach involves collecting a dataset of labeled images, where each image is categorized as either “with mask” or “without mask”. Using this dataset, a model can be trained to recognize patterns and features that distinguish between the two categories. Common algorithms for image classification are CNNs, SVM, Random Forest (RF), K-Nearest Neighbors (KNN), Naïve Bayes, etc.
- Object Detection: Object detection techniques can be employed to locate and classify face masks within an image or video. These models can identify the presence and position of face masks in real-time applications. Currently, for this application, several deep-learning techniques are applicable, like CNNs (e.g., R-CNN-Region-based CNN, Fast R-CNN, Faster R-CNN, YOLO-You Only Look Once, SSD-Single Shot MultiBox Detector, EfficientDet, etc.), which are discussed in Section 3.
- Facial Landmark Detection: Machine-learning models can also be trained to detect facial landmarks, such as the nose, mouth, and eyes, to determine if a face mask is properly worn. By analyzing the spatial relationships between these landmarks, the model can infer the presence and alignment of a face mask. Techniques like the Histogram of Oriented Gradients (HOG) combined with SVM or more modern methods like facial landmark detectors based on deep learning architectures (e.g., OpenPose, DLIB) can be utilized.

Combining various ML prototypes, including Support Vector Machines (SVM), decision trees, and combination techniques, in [23], the authors presented a hybrid deep transmits learning prototype for identifying face masks. The hybrid deep transfer-learning model utilized Resnet 50 feature extraction and three classifiers (i.e., SVM, decision trees, and ensemble approaches). Logistic regression, K-Nearest Neighbors Algorithm, and linear regression are examples of ensemble methods used to construct M-classifiers and train each before combining and averaging their outputs. Real-World Masked Face Dataset (RMFD) accuracy was 99.64%, simulated masked-face dataset accuracy was 99.48%, and accuracy within the untamed dataset was 100%.

SVM is a common classifier for medical applications; it is a supervised machine-learning technique for classification and regression problems. This algorithm suits linear or nonlinear classification applications, outlier identification, regression, and even outlier detection. In detail, SVMs can be used for various tasks, including text classification, image classification, spam detection, handwriting identification, gene expression analysis, face detection, and anomaly detection. SVM is adaptable and powerful in various applications because it can handle high-dimensional data and nonlinear relationships. Nevertheless, its primary application is for classification problems, particularly those involving two classes or binary classification. It employs hyperline or hyperplane to determine to which class new unlabeled data belongs during testing [24]. SVM uses the training subset of data to identify which labels they correspond to, then to build a hyperline for two classes or a hyperplane for more than two classes to distinguish between data.

In [25], the authors introduced a hybrid face mask identification model that combines deep learning, handcrafted feature extractors, and traditional machine learning classifiers. In particular, the proposed approach combines a Random Forest classifier on a hybrid feature set created by CNN and a handcrafted feature extractor from the input pictures to distinguish masks from faces. Principal component analysis, or PCA, is additionally employed for feature selection. Although the system has a test accuracy of about 62% for a random forest with 100 trees, this accuracy could be improved by expanding the training data set and adding historical data sets that contain localized information that may be useful concerning a specific geographic location.

Ensemble methods in machine learning are techniques that combine multiple models to improve overall prediction performance and generalization. The idea behind ensemble methods is that by combining diverse models, their weaknesses can be compensated, resulting in a more robust and accurate prediction. Ensemble methods are widely used in various machine learning tasks and have shown to be highly effective in many real-world applications. Popular ensemble methods include Bagging (Bootstrap Aggregating), Random Forest, Boosting, and Stacking. There are three phases of the ensemble method:

1. Create M classifiers.
2. Train each individual classifier.
3. Combine the M classifiers and calculate their average throughput.

### Datasets for Developing Face Mask Recognition Algorithms

Datasets play a crucial role in developing and training face mask recognition algorithms. It is important to note that the dataset’s quality, diversity, and size directly influence face mask recognition algorithms’ performance and generalization capabilities. Collecting and curating high-quality datasets encompassing a wide range of real-world scenarios is crucial for developing effective and reliable algorithms. Additionally, ongoing efforts to ensure the inclusiveness and fairness of datasets help minimize biases and disparities in algorithm performance. The main publicly available datasets for developing face mask recognition algorithms are summarized in Table 1.

In addition to what was previously reported, Masked Facial Recognition includes bounding boxes for the 853 photos from the three classes in the PASCAL VOC format. The pictures are classified into three different classes: “with a mask”, “without a mask”, and “wrongly worn”. Similarly, the Moxa3K dataset comprises images taken during the epidemic in Russia, Italy, China, and India. There are 3000 total photos in the dataset: 9161 faces without masks and 3015 faces with masks. Furthermore, Real-World Masked Face Dataset contains two data sets: (I) A dataset for masked-face recognition in the real world, obtained by scanning the website pictures; it has 90,000 regular faces and 5000 masks representing 525 individuals after cleaning and labeling. (II) Simulated masked-face recognition datasets comprise 500,000 masked faces from 10,000 people.

Also, LFW (Labeled Faces in the Wild) Simulated Masked Face Dataset is derived from the LFW dataset; this last is constituted by images of famous people gathered from the website. The SMFRD includes the same photos of the LFW dataset to which simulated masks have been applied. The dataset consists of 13,117 faces of 5713 people.

As the previous table shows, the MaskedFace-Net dataset is one of the largest datasets reported in the scientific literature, including images of people with and without face masks of different genders, ages, and ethnicities [26]. This dataset enables the accurate training and testing of machine-learning algorithms, ensuring a high degree of generalization.

## 3. Deep Learning (DL) Techniques Used to Identify Face Masks

**Figure 5:**
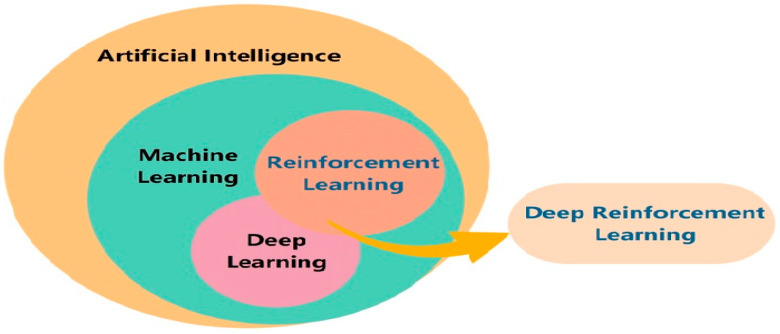
The relation between AI, ML, and DL [37].

AI comprises a variety of technologies designed to mimic the reasoning functions and intelligent behavior of humans. In recent years, DL has been gaining popularity; DL is a subfield of machine learning that focuses on using Artificial Neural Networks to model and solve complex problems. For instance, DL models can identify intricate patterns in images, text, audio, and other data types to generate precise analyses and forecasts. DL techniques can be used to automate processes that ordinarily require human intellect, such as text-to-sound transcription or the description of photographs. As a subfield of AI, ML relies on training algorithms to acquire knowledge and insight from a dataset (Figure 5) [37]. Due to their limitations, ML models can only answer well-managed issues when confronting unstructured or complex problems, unlike DL models, which address unstructured or complex problems. Drawing inspiration from genetic nerve cells, models generated from biological neurons use numerous levels of interpretation to uncover the multidimensional and intrinsic relationship in data. DL techniques can extract significant relationships and dependencies from unstructured or unlabeled information by developing deep hierarchical features in the dataset [38].

Deep neural networks (DNNs) is a term used to describe SE-YOLOv3 (multi-scale object detection network that uses a feature extraction network and multiple detection heads to make predictions at multiple scales) technology occasionally. DL techniques comprise multilayered neural networks in which one or more hidden layers are linked together to form a learning-competent network with complex structures at an elevated level of extraction [39,40].

You Only Look Once (YOLO), ResNet-50, CNN (illustrated in Section CNN for Face Mask Recognition), and Region-based CNN (R-CNN), which extracts a significant amount of region proposals from the input image, identifying their classes and bounding boxes, were considered promising in identifying face marks [41,42]. Also, in [42], DL and ML models were effectively applied to identify COVID-19 from raw data obtained from medical IoT devices. These are only a few examples of the many areas where DL has been successfully applied.

Facial recognition technologies are the optimal solution for insecure systems, such as biometrics or entering a password via a keyboard, because they do not require physical interaction. Nevertheless, using face masks in these systems has presented a significant challenge for artificial vision [43], as half of the face is obscured during facial identification, losing vital information. This issue justifies the obvious need for algorithms to identify a person wearing a face mask [44].

### CNN for Face Mask Recognition

Convolutional neural networks, often called CNNs or ConvNets, are a subclass of neural networks, especially effective in processing input with a grid-like architecture, like images [45]. A digital image is a binary representation of visual data. Each pixel, arranged in a grid-like pattern, has a pixel value to specify how bright and colorful it should be. Similarly to the biological vision system, each neuron in a CNN processes data only in its receptive field. The CNN arranges layers to detect the simpler patterns (e.g., lines, curves, etc.) initially, followed by complex patterns, like faces and objects. They are made up of a variety of building pieces, including convolution layers, pooling layers, and fully connected layers. The convolution layer is the core component of the CNN. This layer produces a dot product between two matrices: the constrained region of the receptive field and the kernel, a set of learnable parameters. The weight-sharing method CNNs uses significantly reduces the number of parameters that must be learned. Translation invariance is also caused by pooling layers and increasing receptive field sizes of neurons in subsequent convolutional layers. The backpropagation process enables iteratively optimizing network weights and biases, minimizing the gradient of the network’s parameters with respect to the loss function [45,46]. In [47], a framework for acquiring distinct features associated with face mask-wearing conditions was proposed. As previously introduced, CNNs are a subset of so-called DL approaches specialized for different problem typologies, like action recognition, inverse imaging problems, and image classification [48,49,50,51].

According to [52,53,54], CNN technologies have been adapted to the needs of humanity over time, resulting in applications in numerous application fields, ranging from agriculture to the military and medicine.

In research settings, neural network analysis has also been applied to the analysis of dental images for diagnostic purposes. Their usefulness and protection should be demonstrated using more precise, replicable, and comparable methods [55].

Although medical imaging modalities such as computed tomography (CT) scans and thoracic X-rays have been used for a long time, they have recently been focused on COVID-19-related applications. A review and discussion of CNN for identifying infected tissues in COVID-19 patients using images obtained from various medical imaging systems can be found in [56,57,58].

Due to recent advancements in CNN architectures for object detection, numerous CNN-based models have demonstrated exceptional performance in detecting face masks. The design of Artificial Neural Networks (ANNs) is replicated using CNN-based prototypes. A classifier is a classification algorithm that accumulates and processes hierarchical characteristics extracted from image data. Thus, input identifiers are assigned to images, and then automatic training is performed, as described in [44,59].

**Figure 6:**
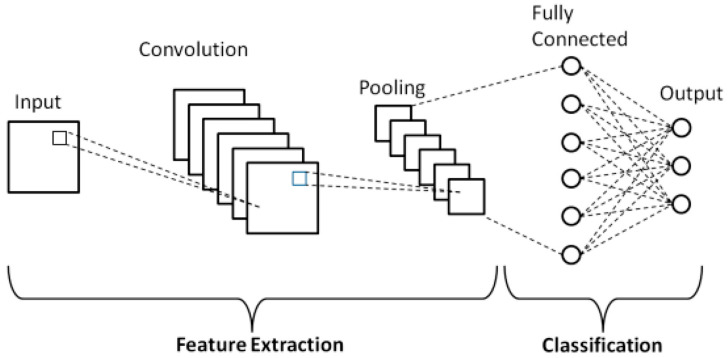
General structure of Convolutional Neural Networks [60].

CNN’s first layer is the input image, followed by the pooling layer, the fully connected layer, and the convolutional layer. The central layer is the convolutional layer, which is responsible for convolving the input image with learnable filters and isolating the resulting image’s characteristics. Neurons designated to identify features in the layer inputs make up each filter. Using small squares of input data, convolution is a technique for learning visual attributes and functioning in conjunction with pixels. The pooling layer reduces the number of neurons in the small rectangular responsive area of the preceding convolutional layer. As proposed in [60], pooling and convolutional layers are responsible for feature extraction (Figure 6).

As previously discussed, CNNs are widely applied in image recognition applications; for instance, the research paper [60] presents a comprehensive solution for image recognition based on CNN, ensuring good classification accuracy (99.5%).

In Ref. [61] a drone was designed for mask detection and social distance monitoring, using Raspberry Pi 4 and convolutional neural network (faster R-CNN) model in order to capture images and detects unmasked persons, respectively. Then, it sends alerts to the people via speaker for maintaining the social distance, and also the detected people details to authorities and the nearest police station.

Using the VGG-16 CNN model, Ref. [62] implements a detection method with a 96% accuracy rate. Similarly, in [63], authors presented the SSDMNV2 model based on the MobileNetV2 architecture, with a 92.64 percent experimental accuracy.

In [64], a 5 × 5 complexity was divided into two 3 × 3 complexities. InceptionV3 is a pre-trained prototype that utilizes handover learning, transferring the trained neural network knowledge to the new prototype in periods of parametric substances. It employs a 48-layer CNN design. Concerning COVID-19 face mask recognition, Ref. [65] proposed a prototype employing transport learning of InceptionV3 transfer learning techniques. The proposed algorithm reached a training accuracy of 99.92% and a test accuracy of 100% using the Simulated Masked Face Dataset (SMFD).

**Figure 7:**
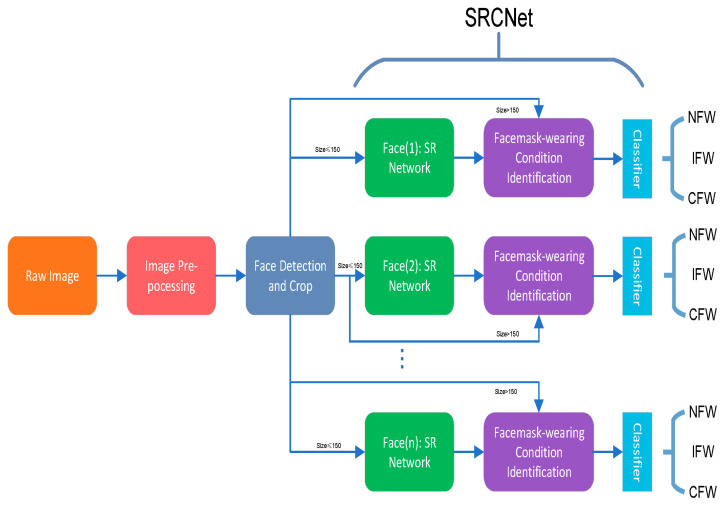
Block diagram of the face recognition algorithm presented in [66], which relies on the SRC Net and face mask-wearing condition identification process.

The authors of [66] propose an automatic face mask recognition method constituted by an image Super-Resolution and Classifier Network (SRCNet), evaluating the performance of a three-category classification algorithm using unrestricted 2D face pictures. The SRCNet was used to identify if masks were worn (Figure 7). The obtained test results demonstrated that the proposed method reached an improved accuracy (98.70%) compared to conventional image classification techniques.

In [67], a face mask identification system employing ocular data and ImageNet-trained CNN was presented. The accuracy obtained ranged between 90 and 95%.

ResNet is an acronym for Residual Networks. ResNet’s benefit was that it enabled the successful training of DNNs with 150 or more layers. Before ResNet, the problem of disappearing constituents made training DNNs extremely problematic. Residual Network employs the concept of skip connection to address the problem of missing ingredients. This accomplishment was made possible by ResNet’s bypass connection feature; as a result, the weight did not decrease to a negligible level [68].

**Figure 8:**
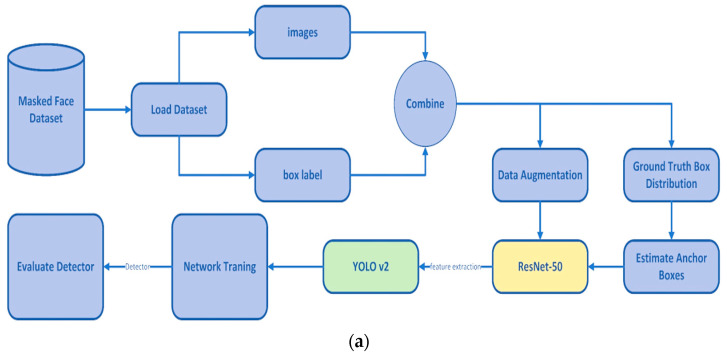

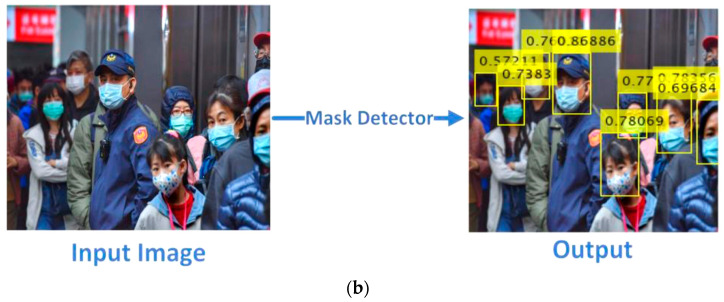
Face recognition model including ResNet50 and YOLO v2 model presented in [70] (**a**); the outcome of the proposed masked-face detector (**b**).

ResNet-50 is a 50-layer CNN applied to derive features. This architecture has been successfully implemented in various disciplines, such as image classification and object recognition [69]. The authors of [70] employed the ResNet-50 model for feature extraction, while YOLOv2 was used to detect medical face masks, achieving an 81% accuracy for face mask precision detection (Figure 8a); also, Figure 8b reports the outcomes of the proposed face mask detector, reported a bounding box for each detected mask and the corresponding score.

A detection system for recognizing unmasked individuals was proposed in [71]. The proposed method comprises three components: the first layer (ResNet-50) used a Feature Pyramid Network (FPN), the second element included a Multi-Task CNN (MT-CNN), and the third element was a CNN classifier to identify masked and unmasked features. The proposed method was implemented on a mobile robot (Thor) and evaluated using a dataset of recordings captured by the robot in public spaces. The tests demonstrated that the proposed system obtained an F1 score accuracy of 99.2%.

In [72], an effective method based on computer vision was proposed, with the authors focusing on real-time automated surveillance of both social distance and face mask perspectives in public spaces. If these conditions are violated, the system will send an alert signal to the authorities.

A model that can detect people without masks using a facial detection system was proposed in [73]; the collected data were integrated with a public recognition database to compile information about the suspect, and a text message was sent to his mobile phone.

In [74], a system that can identify individuals not wearing masks in a smart city network equipped with Closed-Circuit Television (CCTV) cameras to monitor public areas was proposed. The corresponding authority is notified via the city’s network when a person is uncovered. They trained a deep learning architecture using images of persons wearing and not wearing masks. The accuracy of the trained architecture was 98.7%.

In [75], a CNN-based architecture with multiple stages was proposed to identify the individuals not wearing masks. The model’s detection accuracy was 91.2%. A near real-time technique for automatically distinguishing face masks was proposed in [76]. The proposed architecture, in conjunction with CNN, obtained a 95.8% accuracy in recognition.

The dual-stage CNN model proposed in [77] could differentiate between mask-wearing and mask-free individuals and be integrated with pre-installed CCTV cameras. The detection precision of this model is 99.98%.

## 4. Mobile Networks for Face Mask Detection (Mobile Netv1 and MobileNetv2)

In computer vision and deep learning, face mask recognition algorithms have seen a tremendous increase in popularity. The method proposed in [78] employed deep learning frameworks, like Tensor-Flow, Keras, and OpenCV libraries, to identify face masks in real time. The trained MobileNet model generated an accuracy score and F1 score of 99.9%.

Convolutional neural networks, such as MobileNet, are specialized for embedded and mobile vision applications. They are built using depthwise separable convolutions, which are lightweight deep neural networks that can have minimal latency for embedded and mobile devices.

**Figure 9:**
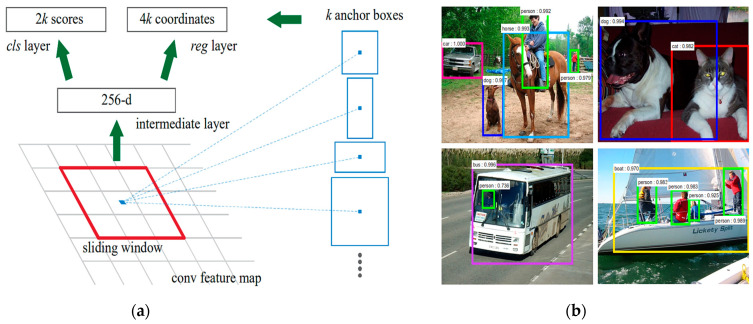
Diagram depicting the operating modalities of the proposed Region Proposal Network (RPN) (**a**) and example of application on PASCAL VOC 2007 test (**b**) [79].

The MobileNets architecture proposed in [79] used depth-wise discrete convolutions to construct Lightweight Region Proposal Networks (RPNs), resulting in 73% accuracy for the PASCAL VOC 2007 trainval (Figure 9).

The smaller footprint and faster performance of MobleNets made them suitable candidates for deep-learning models. MobileNets lacked adequate accuracy compared to other prototypes, such as Accelerated R-CNN and InceptionV2 [80], a drawback of these typologies of the framework.

In [81], a detection architecture employing MobileNetV2 face mask recognition was proposed; the system prototype was developed and tested, demonstrating its correct operation in determining whether or not a person was wearing a mask. Since the proposed model was a lightweight CNN, it can be implemented into both mobile and computer vision techniques.

## 5. Face Mask Detection Sensors

Sensors play an essential role in the fight against COVID-19, as they are utilized in various ways, including detecting people wearing face masks and detecting COVID-19 by measuring a person’s temperature.

**Figure 10:**
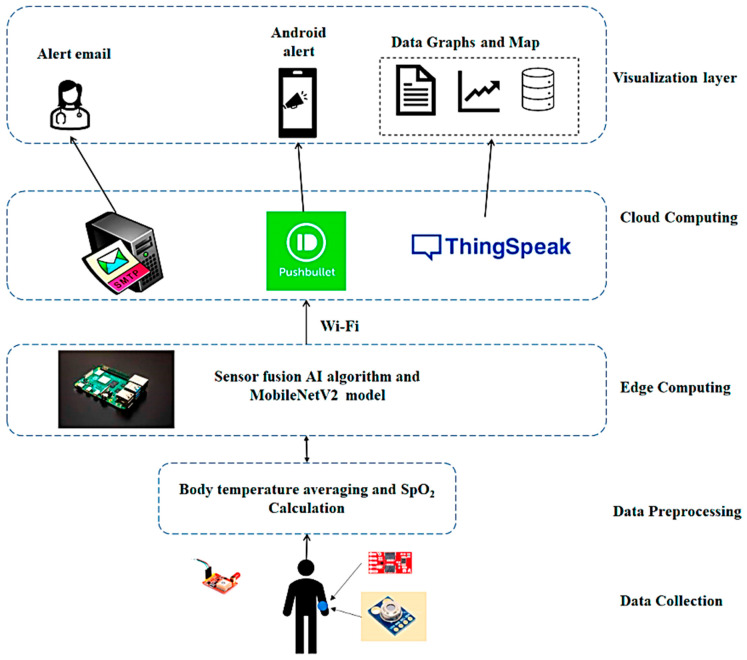
Architecture of the Intelligent IoT sensor presented in [82].

In [82], a novel Sensor Fusion (SF) method for detecting COVID-19 suspects was proposed. Also, the proposed system combines the SF algorithm with the MobileNetV2 model for face mask detection, improving the prediction accuracy. An Arduino board is interfaced with an IR temperature sensor and a PPG (photoplethysmography) sensor to acquire body temperature and SpO_2_. The Arduino forwards biophysical data to a Raspberry Pi board that deploys SF to detect COVID-19 suspects. MobileNetV2 is run on the Raspberry Pi board library to determine suitable mask alignment using images acquired by a camera module (Figure 10). The MobileNetV2 reached a 99.26% accuracy. On a cloud server, health data are perpetually monitored and stored (ThingSpeak). When a COVID-19 suspect is identified, healthcare authorities are notified via email with the infected person’s GPS location.

The Smart Screening and Disinfection Walkthrough Gate (SSDWG) was proposed in [83] to control the entrances to public buildings. The SSDWG is designed to perform rapid screening, which includes measuring temperature with a contactless sensor and preserving the record of infected individuals for increased control and monitoring. The proposed system utilized real-time deep-learning models to detect and classify face masks. This module implemented transfer learning with VGG-16, MobileNetV2, Inception v3, ResNet-50, and CNN models. The presented system achieved a precision of 99.81%.

In [84], the authors proposed an IoT-based system for COVID-19 indoor safety monitoring. It comprises a sensor-based temperature measurement subsystem, a computer vision subsystem for mask detection, and a Raspberry Pi-based social distancing controller. In detail, the non-contact sensor measures the person’s temperature; if the person’s body temperature is higher than normal, the door is locked, and a message comprising the temperature value and location is sent to the server. The subsequent phase is mask detection; a CNN and a deep learning technique are combined for this purpose. The face frame is resized, converted into an array, and pre-processed using the MobileNetv2 algorithm. The following step involves the implemented model to forecast the processed input picture. The video frame will also be labeled with the subject’s mask-wearing status, along with the percentage inferring accuracy. The test results demonstrated that the computer vision subsystem reached 91% accuracy.

In [85], B. Varshini et al. proposed an IoT-enabled smart door that uses a machine-learning model for body temperature monitoring and face mask recognition. In detail, a model employing a real-time deep learning system implemented on Raspberry Pi was implemented to detect face masks and the number of individuals present at any given time. This model was based on a CNN deployed by the TensorFlow software library. The pictures used to train and test the model were from the internet. The dataset comprises 690 pictures with masks and 686 images without masks in this collection. The trained model obtained a 97% accuracy using the face mask detection algorithm.

Similarly, in [86], the authors introduced a face mask detection system to fight the diffusion of the COVID-19 pandemic operating on pictures and videos; furthermore, the system could monitor body temperatures to detect potentially infected people and automatically spray the disinfectant. They explored many classifiers, including the Symbolic Classifier and Support Vector Machine (SVM). In detail, three approaches are used to classify histopathological pictures. The first technique, nuclei segmentation, denotes cellular alterations. The second technique deals with textural characteristics; the last technique relies on variations in color densities. The main feature that characterizes mitotic behavior is its form. The features set and cellular structure of each blob, namely the area, perimeter, solidity, and circularity, are used to extract the morphological differences. Then, the best shape features are extracted, which characterize the behavior of mitosis. Detected nuclei are used to extract texture characteristics. Also, a custom CNN classification algorithm was proposed for face mask detection, including several Neuro-Fuzzy layers. Fifty different image datasets were tested in various experiments to evaluate their performance. The test results demonstrated that the proposed CNN method reached 91.11% accuracy with 7.24 s inferring time.

**Figure 11:**
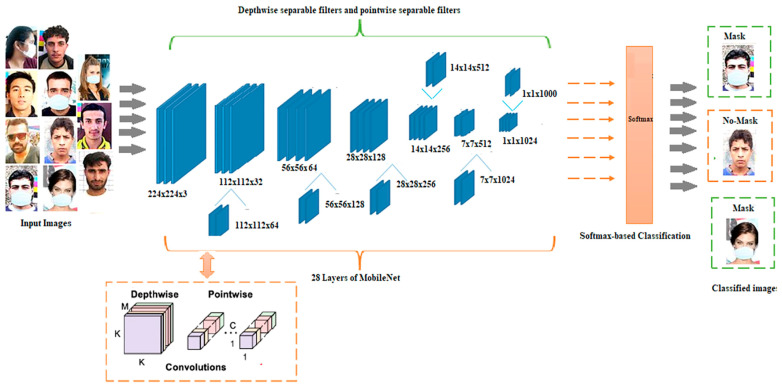
Architecture of masked-face recognition algorithm based on DWS-based MobileNet proposed in [87].

Finally, a DWS-based MobileNet, a Depthwise Separable Convolution Neural Network, was introduced in [87] (Figure 11). Instead of using 2D convolution layers, the suggested network uses depth-wise separable convolution layers, ensuring fast training with fewer parameters.

It comprises a 1 × 1 convolution output node where each pulse’s spatial convolution is carried out separately. A one-dimensional maximum on the output of the Rectified Linear Unit (ReLU) activation function was employed, supplied by the output of the convolution layer. While the pooling layer’s filter size is fixed at 20 with a step number of 2, the first convolutional layer’s filtering size and depth are both modified to 60. The convolution layer’s output for the fully connected layer input is flattened down to a stepping of six. They use a dropout method in which neurons are randomly turned off during training to prevent overfitting. The Moxa3K dataset was employed for this investigation, comprising 3000 photos, 2800 used for training, and 200 for testing. The test results indicated that the DWS-based model has a greater accuracy (93.21%) than SVM and CNN, using the AIZOO FACE MASKS dataset.

Finally, Table 2 summarizes the main research lines investigated in this review work, reporting the main advantages and weaknesses of each research, as well as the current approaches to solving the problems.

## 6. Challenges in Face Mask Recognition Systems

Face mask recognition has been criticized for its precision, reliability, and improper use of private information. In detail, listed below are some of the obstacles associated with masked-face recognition [88,89]:

- Accuracy: Face mask recognition technology should be developed and tested rigorously to ensure high accuracy rates, particularly in identifying both masked and unmasked individuals. False positives, where individuals are incorrectly identified as not wearing masks, can have severe consequences, such as denying access to essential services or causing unnecessary alarm. No face mask-recognition algorithm reaches 100% accuracy, even with the most sophisticated software. However, the technology is generally considered satisfactory, with at least 98% accuracy rates.
- Mask Variability: Face masks come in various shapes, sizes, colors, and designs. Recognizing and accommodating the diverse range of masks can be challenging for the algorithms. Each mask type may introduce unique textures, patterns, or features that must be considered for accurate recognition.
- Lighting and Environmental Factors: Variations in lighting conditions, such as shadows, reflections, or poor illumination, can affect the visibility of facial features and the overall performance of face mask recognition algorithms. Challenging lighting conditions can decrease accuracy and introduce additional variability.
- Rapid Deployment and Adaptation: The need for face mask recognition arose rapidly during the COVID-19 pandemic, requiring quick deployment of technology. Developing robust algorithms and adapting them to different scenarios and environments can be challenging due to the limited research, testing, and optimization time.
- Computational Resources: Implementing real-time face mask recognition systems that quickly process large amounts of data can be computationally demanding. High-speed processing and response times are crucial for applications where real-time identification is required.
- Database necessity: Training accurate and unbiased face mask recognition models requires diverse and representative datasets, including individuals wearing different types of masks. The availability of such datasets, as well as potential biases present in the data, can impact the performance and fairness of the algorithms.
- Ethical and Privacy Concerns: Face mask recognition involves capturing and processing personal biometric data, raising concerns about privacy, consent, and potential misuse of the collected information. Ensuring robust data protection measures, transparency, and addressing privacy concerns are important for the ethical use of the technology.
- User Acceptance and Cooperation: Face mask recognition systems often require user cooperation, such as proper positioning of masks, removing obstructions, or following specific guidelines. Achieving widespread user acceptance and compliance can be challenging, impacting the overall effectiveness of the technology.

Image variations compared to the factors discussed above (i.e., mask variability, positioning, lightning, poses) make it more difficult for the face mask recognition algorithm to detect the presence of a face mask. Indeed, it can be more difficult to compare two images if there are significant differences in head position, light orientation and intensity, mask typology, etc. There are two options for addressing these issues:

- Use numerous forms of training sets to acquire knowledge.
- The use of deep learning techniques facilitates the correction of these differences.

Addressing these challenges through ongoing research, advancements in computer vision, machine-learning techniques, and contemplating ethical implications can contribute to developing more accurate, reliable, and responsible face mask recognition systems.

## 7. Results and Discussions

In this section, the previous scientific works are compared and further analyzed to bring out trends and guidelines for developing modern face recognition systems.

As discussed above, face recognition employs numerous classification and processing methods, including but not limited to DL and ML algorithms, as well as Mobile Networks (V1 and V2). Table 3 summarizes the prototype discussed in the previous sections, classifying them from the perspective of the employed technique, implemented methodologies, application purposes, and considered domain.

As shown in Table 3, CNNs are the most diffused tool for face mask and face-masked recognition detection, given the several offered advantages like spatial invariance, parameter sharing, translation invariance, and scalability [90,91,92]. As discussed above, CNNs offer a powerful framework for extracting and learning discriminative features from images, making them well-suited for masked-face recognition, face mask detection, and other computer vision tasks. Their ability to learn hierarchical representations, handle spatial variations, and scale to large datasets justifies their effectiveness and widespread use in computer vision applications.

Furthermore, the MobileNets are gaining ground in recent years thanks to their lightness and efficiency, making them ideal for implementations on mobile and embedded devices with limited computational resources. MobileNet architectures can independently process and capture unique features from sensor data, identifying complex relationships and dependencies, thus improving performances and reducing latency [93,94]. Also, deep learning, machine learning, and mixed MobileNet-sensors algorithms are common solutions for deploying masked-face recognition and face mask detection algorithms.

Afterward, the performance of the techniques previously discussed is evaluated to determine the most promising and performant solution for developing the future masked-face recognition system. Table 4 summarizes the research works on facial mask detection using deep learning and CNN, as well as the accuracy of the proposed techniques.

As shown in Table 4, surveyed algorithms varied from the prototype model/domain. Deep learning uses techniques such as public recognition database to compile information, a Hybrid deep transfer learning model, TensorFlow, Keras, and OpenCV; they also differ in the achieved accuracy from 95% to 99.64%. On the other hand, CNN combined with deep learning or consisting of multi-stage achieved better accuracy than DL with 99.98%. Face recognition sensor-based networks achieved an accuracy range from 91% to 99.81%. The lowest achieved was for Lightweight Region Proposal Networks (RPNs) (73%), compared with other techniques falling within this range.

MobileNetV2 is known for its improved accuracy compared to the original MobileNetV1 architecture. The accuracy of MobileNetV2 can vary depending on the specific dataset, task, and training configuration. However, in general, MobileNetV2 has demonstrated competitive performance on various computer vision tasks, including image classification [77,81].

Similarly, hybrid approaches that combine DL and classical ML classifiers [23] are valuable solutions in terms of performance (99.64% accuracy) but suffer in terms of processing (i.e., training and inferring) time and required resources. This result is predictable since, by stacking different algorithms, higher computational requirements are required.

## 8. Conclusions

Although the effects of the COVID-19 pandemic have diminished recently, it still affects various regions of the globe. According to the World Health Organization, social isolation and the use of face masks are two of the most essential methods to contain the pandemic’s spread. Recently, facial recognition has been the subject of several international studies. The presented review is focused on the most recent findings reported in the scientific literature regarding methodologies and systems for masked-face recognition and face mask detection developed to fight the COVID-19 pandemic. At first, classical ML algorithms and models for masked-face recognition and face mask detection are reviewed and discussed, lingering on solutions combining multiple ML models to improve their performance. After, DL techniques used to identify face masks are presented, focusing on CNN applications. Furthermore, modern tools, like Mobile Networks (MobileNetv1 and MobileNetv2), are introduced for facial recognition and face mask detection applications. After, IoT-based sensors for fighting the COVID-19 pandemic diffusion are reviewed; based on ML and DL algorithms, these prototypes enable rapid screening of numerous people, assessing whether they wear masks and body temperature. Then, challenges in facial recognition are reported, along with a comprehensive comparative analysis and discussion for outlining the features of face recognition systems.

In conclusion, in recent years, there has been a rise in the use of computer vision for masked-face recognition and face mask detection; to investigate their perspectives, we analyzed and compared modern facial recognition techniques, focusing on their performance. From the presented analysis, MobileNetV2 is the best candidate for designing the next generation of face recognition systems, given their performance, memory requirements, training time, etc. [95]. Likewise, hybrid approaches combining both DL and classical ML classifiers are worthy solutions in terms of performance (99.64% accuracy) [23]; however, they experience issues in terms of processing (i.e., training and inferring) time and required resources. Finally, we analyzed IoT-based sensors for face mask detection, demonstrating that they are effective tools for fighting the future pandemic, given their ubiquity and performance.

## Figures and Tables

**Table 1:** Table summarizing the main datasets to develop face mask recognition algorithms.

Dataset	Total Number of Images	Number of Images with Mask	Number of Images without the Mask	Method	Mean Average Precision (%)
MaskedFace-Net [26]	133,783	67,049	66,734	Haar-features + Face and Nose Detection algorithm	99.92 [27]
FMLD [28]	41,934	29,532	33,540	FetinaFace AntiCov	92.93 [29] 88.91 [30]
ISL-UFMD [31]	21,816	10,698	10,618	Inception-v3	98.20 [31]
Face Mask Detection [32]	7553	3725	3828	CNN	98.00 [33]
MDMFR [34]	6006	3174	2832	DeepMaskNet	100.00 [19]
BAFMD [35]	13,000	6264	3118	YOLO-v5 AntiCov	86.80 [35] 78.10 [35]
MAFA [36]	35,806	911	30,811	YOLO-v5 AntiCov	87.30 [35] 84.90 [35]

**Table 2:** Summarizing table of the main discussed research lines.

Recognition Techniques	Technology	Description	References
Machine Learning	Support Vector Machines (SVM), decision trees, Multilayered Deep Neural Networks DL used for Access Control, Convolutional-neural-network-based action recognition	These cutting-edge techniques prioritize accuracy in some situations and speed in others. This section describes object detection utilizing the deep learning approach instead of the benefits of deep learning techniques in a real-time application.	[24,37,38,39,40,41,42,43,44,47,48,49,50,51,52,53,54,55,56,57,58]
Computer vision	There are now numerous object detection methods available.	For object detection, it is possible to find and recognize specific kinds of things in pictures and videos. Additionally, this method localizes the objects in the supplied image using bounding boxes. This can count the number of items in the image that has been provided.	[14,15,16]
CNN	Artificial Neural Networks, Layered CNN Inceptionv3 Super-Resolution of Images (SRCNet) Residual Networks	They focus on areas, or regions, in a photo similar to other areas, such as the pixelated region of an eye. If this region of the eye matches up with other eye regions, then the R-CNN knows it has found a match. However, CNNs can become so complex that they “overfit,” which means they match regions of noise in the training data and not the intended patterns of facial features.	[44,47,48,49,50,51,52,53,54,55,56,57,58,59,60,61,62,63,64,65,66,67,68,69,70,71,72,73,74]
Mobile Networks (MobileNet v1 and MobileNetv2)	Deep learning, TensorFlow, Keras, and OpenCV	MobileNets V1 are built on a simplified design that creates lightweight deep neural networks using depth-wise separable convolutions. Building on the concepts of MobileNet V1 MobileNet V2 employs depth-wise separable convolution as effective building pieces. Linear bottlenecks between layers and short connections between bottlenecks are two new characteristics added to the architecture by V2.	[78,79,80,81]
Sensors	Sensor Fusion (SF) approach with MobileNetv2, deep learning	Fusing data from at least two sensors is known as Sensor Fusion. Perception is the analysis and classification of sensor data to find, recognize, categorize, and track objects (e.g., faces).	[82,83,84,85,86]

**Table 3:** Face recognition techniques and prototypes.

Technique	Prototype/Method/Domain	Ref.
Machine Learning	Support Vector Machines (SVM), decision trees, and combination techniques	[23]
	SVM	[24]
Deep Learning	Multilayered Deep Neural Networks	[39]
	DL used for Access Control	[42,43]
Convolutional Neural Networks	A framework consideration module.	[44]
	Convolutional-neural-network-based action recognition	[47,48,49,50,51]
	CNN adapted in the agriculture, defense, and medicine sectors.	[52,53,54,55,56,57,58]
	Artificial Neural Networks	[44,54]
	Layered CNN	[60,61,62,63,76,77]
	Inceptionv3	[64,65]
	Super-Resolution of Images (SRCNet)	[66,67]
	Residual Networks	[68,69,70,71]
	Model for detecting people don’t wear masks	[72,73,74]
Mobile Networks (MobileNet v1 and MobileNetv2)	Deep learning, TensorFlow, Keras, and OpenCV	[78,79,80,81]
Sensors	Sensor Fusion (SF) approach with MobileNetv2, deep learning	[82,83,84,85,86]

**Table 4:** Achieved accuracy for COVID-19 facial mask detection techniques.

Work	AI Models	Achieved Accuracy
M. Loey et al., (2021) [23]	Hybrid deep transfer learning model Support Vector Machines (SVM), decision trees, and combination techniques	99.64%
M. Loey et al., (2021) [70]	A model that integrated YOLO-v2 and ResNet-50 DL (Residual Networks)	81%
M. M. Rahman (2020) [74]	Lightweight neural network (for detecting people who do not wear masks)	85%
M. Inamdar and N. Mehendale (2020) [73]	A novel DL model (utilizing public recognition database to compile information)	98%
S. Yadav (2020) [72]	Deep Learning and computer vision-based approach (method based on computer vision)	95%
T. Rao and S. Devi (2020) [75]	Multi-stage CNN architecture for face mask Identification.	91.2%
H. Lin et al., (2021) [76]	CNN combined with the DL approach for mask identification.	95.8%
A. Chavda et al., (2021) [77]	Multi-stage CNN architecture for face mask detection	99.98%
S. E. Snyder et al., (2021) [71]	Different types of deep learning for detecting face mask ((ResNet-50) with Feature Pyramid Network (FPN), Multi-Task CNN (MT-CNN), CNN classifier)	99.2%
S. Taneja et al., (2021) [81]	CNN-based mask identification Method Utilizing OpenCV and MobileNetV2	99%
G. H. Christa et al., (2021) [78]	Deep learning, TensorFlow, Keras, and OpenCV	99%
S. Ren et al., (2015) [79]	Lightweight Region Proposal Networks (RPNs)	73%
R. K. Shinde et al., (2022) [82]	Sensor Fusion (SF) approach	99.26%
S. Hussain et al., (2021) [83]	Smart Screening and Disinfection Walkthrough Gate (SSDWG)	99.81%
N. Petrović and Đ. Kocić (2020) [84]	Contactless sensor with computer vision	91%
B. Varshini et al., (2021) [85]	Sensors with deep learning	97%

## Data Availability

Data from our study are available upon request.
